# Surgical cure of intractable epilepsy caused by retained intracranial foreign body under cortical electroencephalography monitoring: case report and literature review

**DOI:** 10.3389/fsurg.2025.1614564

**Published:** 2025-06-06

**Authors:** Zhigang Tan, Ming Wang, Jingwei Wan, Xinxin Li, Yan Cui

**Affiliations:** Department of Neurosurgery, The Second Xiangya Hospital of Central South University, Changsha, Hunan, China

**Keywords:** epilepsy, retained intracranial foreign body, EEG, neurosurgery, post traumatic epilepsy

## Abstract

Post-Traumatic Epilepsy is a common and severe complication following brain trauma. However, there are few reports on intractable epilepsy caused by retained foreign bodies. This article reports a case of a patient with a metal foreign body retained in the cranium for 20 years due to trauma, resulting in drug-resistant epilepsy which type is focal to bilateral tonic-clonic seizure. Through preoperative imaging and neurophysiological techniques, the foreign body and epileptogenic zone were precisely located. Intraoperative cortical electroencephalography monitoring was used to accurately remove the foreign body and resect the epileptogenic focus, which pathological result indicates gliosis and calcification. The intractable epilepsy was effectively controlled, and the patient experienced no seizures postoperatively.

## Introduction

Post-Traumatic Epilepsy (PTE) is a common and severe complication following traumatic brain injury, with its incidence varying depending on the type and severity of the trauma. However, there are few reports in the literature on intractable epilepsy caused by retained foreign bodies after cranial trauma (Table 1). We performed operation to remove residual intracranial metal foreign body that had remained in skull for 20 years, curing the patient's refractory epilepsy.

**Table 1:** Summary of literature reports on intracranial foreign bodies and epilepsy.

Author	Journal	Type of foreign body	Source of foreign body	Patient demographics	Seizure manifestation	Treatment	Clinical outcome
Ullah, Z. et al. (1)	Cureus	Ring-shaped metal	Trauma? Child abuse?	14-year-old, male	Generalized tonic-clonic	Refused surgery; drug control (sodium valproate)	Unclear
Saeidiborojeni, H. R. et al. (2)	J Res Med Sci	Cotton fiber	Retained from brain tumor surgery 19 years ago	45-year-old, female	Grand mal seizures	Surgical removal	Seizure control
Chao-bin Wang et al. (3)	J Korean Neurosurg Soc	Bullet	Air gun through orbit	40-year-old, male	Grand mal seizures	Surgical removal	First surgery failed; second surgery (1 year 9 months later) controlled seizures
Servet Inci et al. (4)	Neurosurgery	Glass fragment	Suspected glass cup fragment 33 years ago	39-year-old, male	Sleep seizures, grand mal	Surgical removal	Seizure control
Nina Brawanski et al. (5)	Surg Neurol Int	Metal foreign body	Denied trauma	67-year-old, male	Aphasia and grand mal	Surgical removal	Seizure control
Roodrajeetsing Gopaul et al. (6)	Chinese Neurosurgical Journal	Metal sewing needle	Denied trauma	25-year-old, female	Absence seizures	Surgical removal	Seizure control
Qian Chunhua et al. (7)	J Craniofac Surg	Pencil lead	Accidental penetration 30 years ago	40-year-old, male	Grand mal seizures	Surgical removal	Seizure control
Yürekli, V. A. et al. (8)	Seizure	Metal needle	–	52-year-old, female	Sleep seizures, tonic-clonic	Conservative drug therapy	Unclear

## Case presentation

A 39-year-old male was stabbed in the head with a “chisel” near the left eye 20 years ago. Due to limited medical resources at that time, emergency surgery was performed to remove the exposed end of the foreign body, followed by debridement and suturing. Fortunately, the patient did not develop cerebral hemorrhage, infection, brain contusion, or eye injuries. After recovery, the patient returned to normal life. In 2015, the patient experienced sudden loss of consciousness and tonic-clonic seizures of all limbs without obvious triggers, with each episode lasting approximately 3–5 min, followed by gradual recovery of consciousness. Initially, seizures occurred 4–5 times per year. The hospital prescribed sustained-release sodium valproate tablets, but seizure control was poor. In the past 3 years, seizures became more frequent, occurring more than 5 times per month, often preceded by a “premonition”. Multiple hospital visits and treatments with sodium valproate, levetiracetam, and lacosamide failed to significantly reduce seizure frequency.

**Figure 1:**
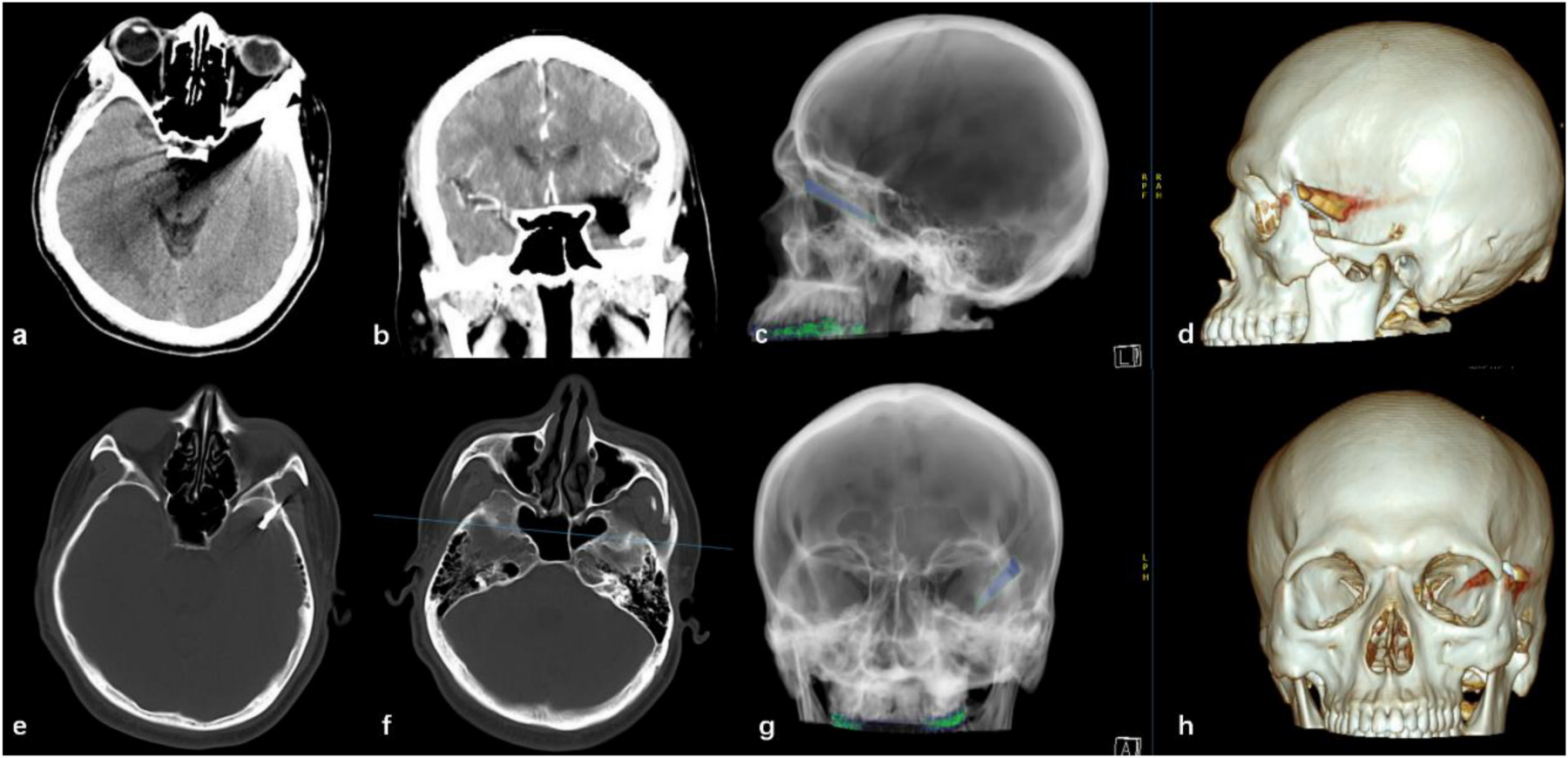
**(a)** Axial CT scan showing a foreign body penetrating the lateral orbital wall into the cranial cavity; **(b)** enhanced coronal CT showing the penetrating foreign body located in the left temporal lobe, at a distance from the sylvian fissure vessels; **(e,f)** axial CT bone window images showing a conical, elongated foreign body penetrating the temporal bone into the cranial cavity, with a fracture at the left zygomatic bone penetration site; **(c,d,g,h)** three-dimensional reconstruction of the intracranial foreign body and skull, showing anterior and lateral relationships.

**Figure 2:**
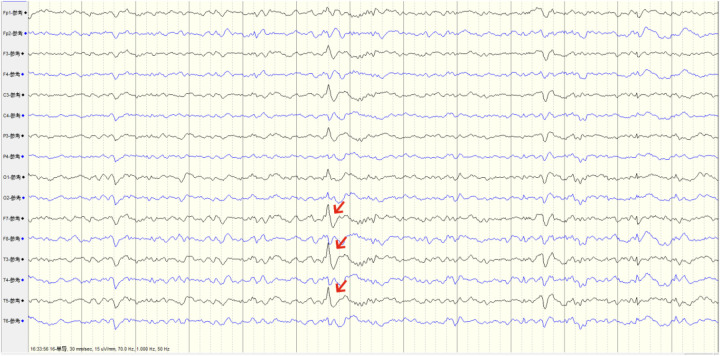
Preoperative EEG capturing abnormal origin discharges from the left temporal pole, consistent with the foreign body penetration site.

**Figure 3:**
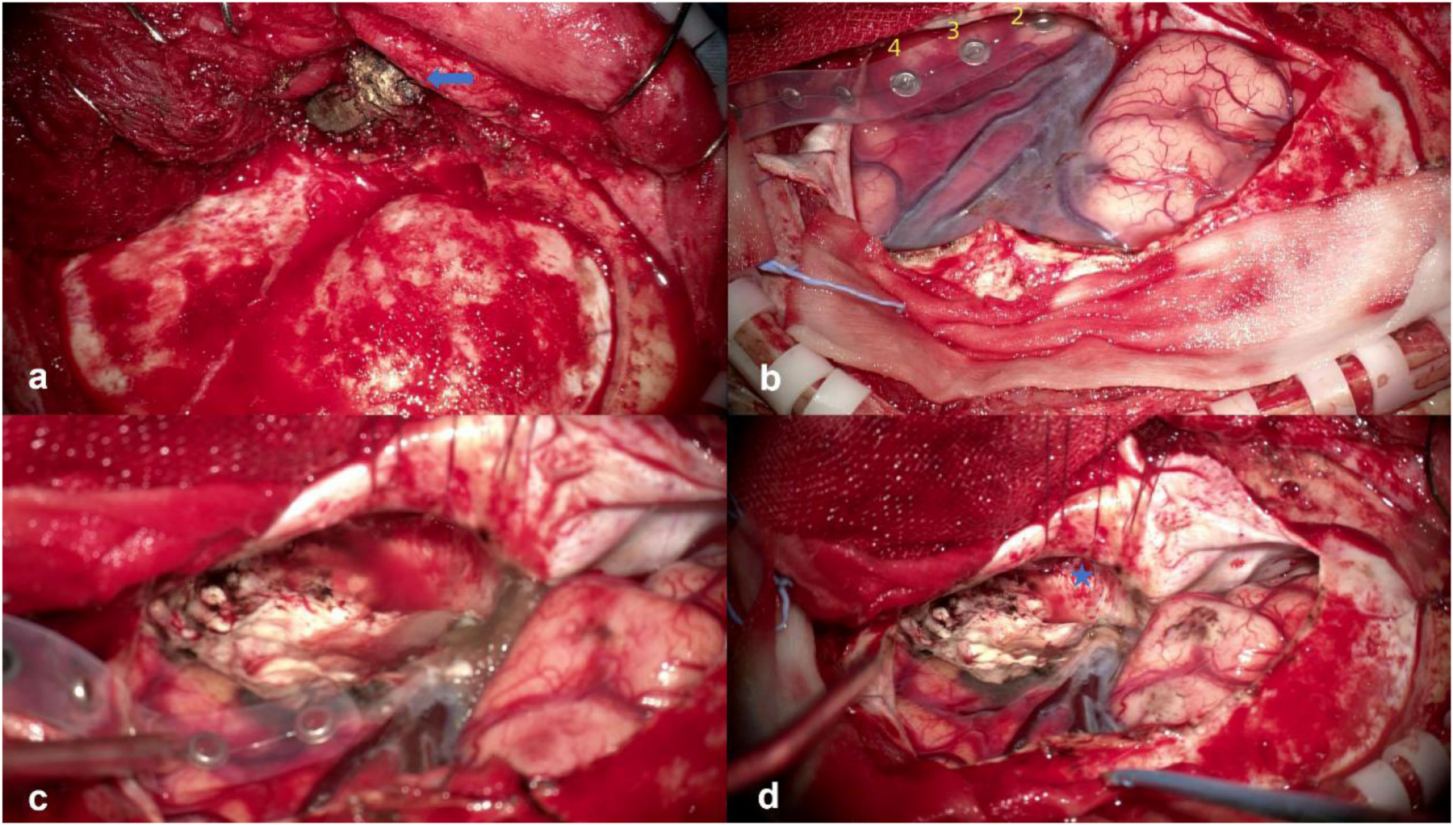
**(a)** After flipping the temporal muscle flap, a metal foreign body (blue arrow) was seen parallel to the lateral orbital wall, penetrating the temporal base bone into the cranial cavity; **(b)** foreign body penetrating the temporal pole, with cortical EEG monitoring electrodes placed on the temporal lobe; intraoperative EEG mapping identified clustered spikes at markers 2, 3, and 4; **(c)** after resection of the abnormal epileptogenic focus, repeat cortical EEG mapping around the margin showed disappearance of abnormal spikes; **(d)** resection of the temporal pole epileptogenic focus exposed the skull base penetration point of the foreign body (blue star).

**Figure 4:**
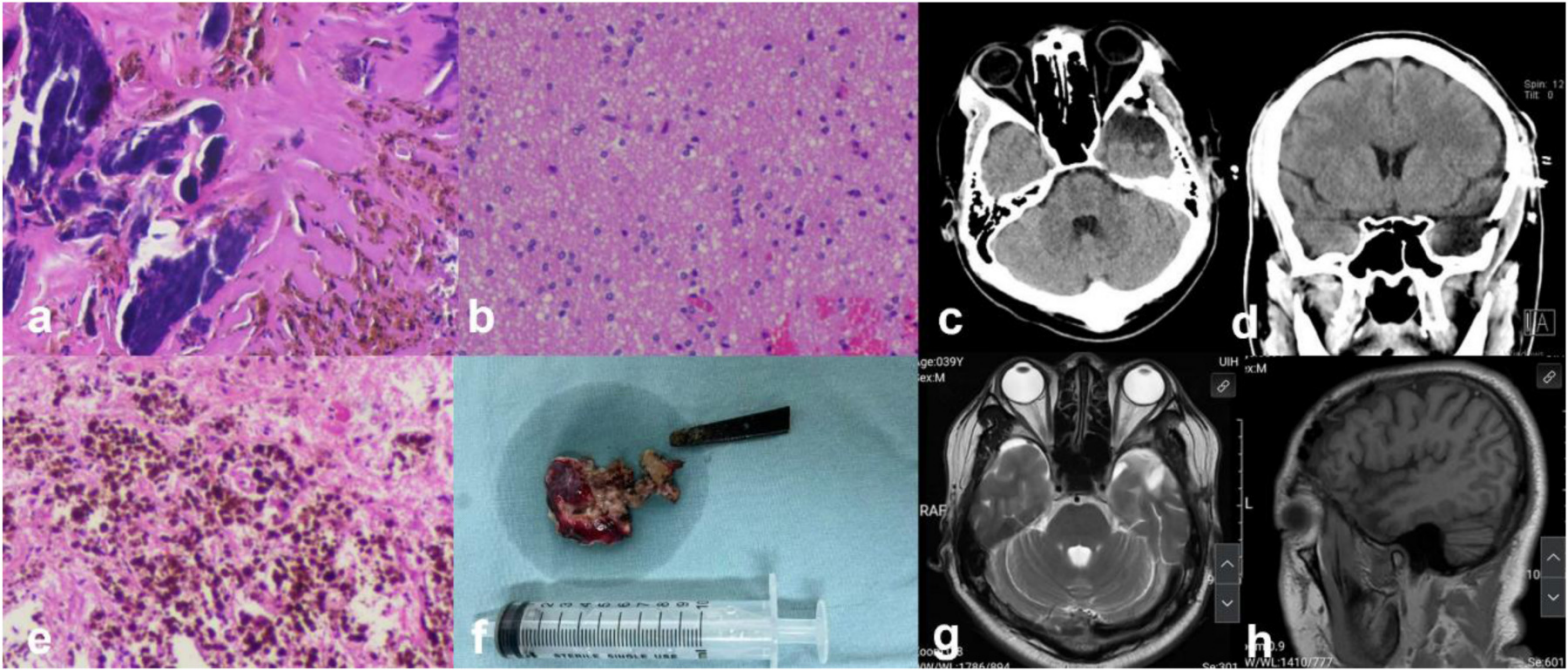
**(a,b,e)** Histopathology (HE*100) showing focal gliosis and calcification, with some areas containing hemosiderin deposits; **(f)** gross view of the removed intracranial metal foreign body and resected epileptogenic focus; **(c,d)** postoperative CT scans (axial and coronal) showing clear removal of the foreign body and postoperative changes after operation; **(g,h)** postoperative 4 month MR showing changes in the temporal pole.

After admission, a head and skull base three-dimensional CT scan revealed a conical foreign body closely adhering to the lateral orbital wall, penetrating into the cranial cavity toward the temporal base (Figure 1). MRI could not be performed due to the suspected metallic foreign body. Preoperative EEG indicated that abnormal epileptiform discharges originated from the left temporal region, closely matching the site of foreign body penetration (Figure 2). According to the updated epilepsy classification published by the International League Against Epilepsy (ILAE) in 2025 (9), the patient's seizure type is focal to bilateral tonic-clonic seizure. Preoperative patient's MoCA (Beijing Version) and MMSE scores were 24 and 26, respectively. Surgery was scheduled, involving microscopic removal of the intracranial foreign body and epileptic focus resection under cortical EEG monitoring. Preoperative neurophysiological monitoring and intraoperative cortical EEG monitoring were prepared. A left pterional approach was used, and upon separating the temporal muscle to its origin, a black, elongated conical foreign body was observed adhering closely to the left lateral orbital wall, penetrating the zygomatic bone and temporal base bone into the cranial cavity (Figure 3). A craniotomy was performed around the foreign body using a milling cutter, preserving the surrounding bone. The bone around the foreign body was thinned with a drill and gradually removed with bone rongeurs, revealing the foreign body penetrating the dura mater, surrounded by proliferated soft tissue, some of which had hardened and calcified. After incising the dura, microscopic exploration showed the foreign body surrounded by cortical tissue. Adhesions between the cortex and foreign body, caused by gliosis, were carefully dissected. Abnormal proliferative blood vessels were coagulated, and adhesions were sharply released, allowing successful removal of the foreign body. Electrodes were placed at the temporal pole cortex, and cortical EEG showed abnormal epileptiform spike discharges at markers 1, 2, 3 and 4. Cortical mapping of the frontal lobe indicated scattered discharges in the middle and inferior frontal gyri. Under the microscope, based on the abnormal discharge area in the temporal pole and avoiding the language area, resection of the temporal pole cortex was performed, successfully removing both the retained intracranial foreign body and epileptogenic focus. The patient was instructed to continue to take levetiracetam 500 mg q12h regularly after operation. He had a follow-up MRI (Figure 4) and short-term (4-h) dynamic EEG at local hospital 4 months after operation, indicating abnormal sharp waves were not detected. The patient was followed up for over 4 months without any seizure recurrence and has returned to normal work.

## Discussion

PTE varies in incidence from 5% to 30% to the complexity of trauma severity, type, and associated conditions (10). Studies have shown that up to 22% of severe traumatic brain injury patients exhibit subclinical epileptic abnormalities on EEG (11). Retained intracranial foreign bodies represent a special type of cranial trauma, which are relatively rare. Previous literature reported that the proportion of seizures in such cases increased (12).

Retained intracranial foreign bodies typically result from penetrating brain injuries or iatrogenic retention during craniotomy. They can lead to various neurological complications, including headaches, focal neurological deficits, intracranial infections and abscesses, or mass effects from inflammatory proliferation and scarring. In this case, the metal foreign body had remained in the cranium for over 20 years, with no significant epileptic symptoms initially. However, over the past decade, seizures became increasingly frequent, progressing focal to bilateral tonic-clonic seizure. Preoperative EEG confirmed that abnormal epileptiform discharges originated from the cortex surrounding the foreign body. After confirming the diagnosis and surgical indications, treatment with epileptic focus resection and foreign body removal under cortical EEG monitoring resulted in significant postoperative seizure control.

The mechanisms by which intracranial foreign bodies cause epilepsy are multifaceted, including mechanical stimulation, inflammatory responses, and scar formation (13). Mechanical stimulation may directly damage brain tissue, triggering abnormal neuronal discharges. Studies suggest that increased neuronal excitability after traumatic brain injury is a key factor in epilepsy development (14). In this patient, the long-term presence of a metal foreign body likely triggered local inflammatory responses and metal ion release, stimulating surrounding glial cell proliferation and focal calcification or fibrosis, forming granuloma-like proliferation. This local gliosis and granuloma formation disrupted normal neuronal network conduction, leading to an imbalance between local excitability and inhibition or the formation of abnormal discharge networks, resulting in drug-resistant epilepsy. Penetrating brain injuries from foreign bodies can also disrupt the blood-brain barrier locally, promoting the aggregation of inflammatory chemokines and further recruiting and migrating inflammatory cells (15). Previous studies have identified CXCL8 and CCL2 as key chemokines mediating neutrophil and monocyte migration to injury sites (16).

Preoperative EEG monitoring to localize epileptogenic foci typically involves standard EEG and invasive stereo-EEG (SEEG). EEG to identify the origin of epileptic discharges is closely related to surgical planning and postoperative seizure recurrence rates (17). Similar to this case, when preoperative EEG and imaging findings are consistent, resection of the lesion or removal of the foreign body can effectively control secondary epilepsy. Intraoperative ElectroCorticoGraphy (ECoG) involves real-time recording of cortical electrical activity during surgery to identify cortical epileptogenic foci and map functional areas such as language and motor regions to avoid damage. In complex, non-standard epilepsy surgeries, studies show that 77.5% of patients using ECoG were seizure-free one year postoperatively, compared to only 37.5% in those without ECoG, indicating that ECoG significantly improves surgical outcomes (18, 19). In this case, after exposing the left temporal lobe cortex, cortical EEG mapping around the temporal pole identified the primary abnormal discharge sites. Microsurgery was used to remove the penetrating foreign body, clear surrounding inflammatory lesions, and resect the abnormal discharge cortex while protecting the left temporal lobe language area. Post-resection cortical EEG mapping confirmed the disappearance of abnormal spikes. Previous reports suggest that confirming the absence of abnormal spike-and-wave signals at the surgical margin via cortical mapping can verify surgical success and predict long-term seizure freedom postoperatively (20). Early studies by the Cleveland Functional Neurosurgery Team on 47 patients with structural brain lesions and drug-resistant epilepsy demonstrated that precise resection of the epileptogenic focus is closely related to seizure control, and even partial, targeted resections can improve postoperative outcomes (21).

## Conclusion

Retained intracranial foreign bodies can lead to secondary intractable epilepsy through complex pathophysiological mechanisms, involving mechanical stimulation, local inflammatory responses, and gliosis or granuloma formation, resulting in heterogeneous brain electrical networks, increased local discharge circuits, and drug-resistant seizures. When preoperative imaging and EEG diagnosis confirm anatomical consistency between the foreign body and the origin of abnormal electrical potentials, combined with intraoperative ECoG, safe removal of the foreign body and resection of the epileptogenic focus can effectively control seizures.

## Data Availability

The original contributions presented in the study are included in the article/Supplementary Material, further inquiries can be directed to the corresponding author.
